# An interpretable radiomics model to select patients for radiotherapy after surgery for WHO grade 2 meningiomas

**DOI:** 10.1186/s13014-022-02090-7

**Published:** 2022-08-22

**Authors:** Chae Jung Park, Seo Hee Choi, Jihwan Eom, Hwa Kyung Byun, Sung Soo Ahn, Jong Hee Chang, Se Hoon Kim, Seung-Koo Lee, Yae Won Park, Hong In Yoon

**Affiliations:** 1 Department of Radiology, Research Institute of Radiological Science and Center for Clinical Imaging Data Science, Yongin Severance Hospital, Yonsei University College of Medicine, Seoul, Republic of Korea; 2 Department of Radiation Oncology, Yongin Severance Hospital, Yonsei University College of Medicine, Seoul, Republic of Korea; 3 Department of Computer Science, Yonsei University, Seoul, Republic of Korea; 4 Department of Radiation Oncology, Yonsei Cancer Center, Heavy Ion Therapy Research Institute, Yonsei University College of Medicine, 50-1 Yonsei-ro, Seodaemun-gu, Seoul, 03722 Republic of Korea; 5 Department of Neurosurgery, Yonsei University College of Medicine, 50-1 Yonsei-ro, Seodaemun-gu, Seoul, 03722 Republic of Korea; 6 Department of Pathology, Yonsei University College of Medicine, Seoul, Republic of Korea

**Keywords:** Magnetic resonance imaging, Meningioma, Radiomics, Radiotherapy, Prognosis

## Abstract

**Objectives:**

This study investigated whether radiomic features can improve the prediction accuracy for tumor recurrence over clinicopathological features and if these features can be used to identify high-risk patients requiring adjuvant radiotherapy (ART) in WHO grade 2 meningiomas.

**Methods:**

Preoperative magnetic resonance imaging (MRI) of 155 grade 2 meningioma patients with a median follow-up of 63.8 months were included and allocated to training (n = 92) and test sets (n = 63). After radiomic feature extraction (n = 200), least absolute shrinkage and selection operator feature selection with logistic regression classifier was performed to develop two models: (1) a clinicopathological model and (2) a combined clinicopathological and radiomic model. The probability of recurrence using the combined model was analyzed to identify candidates for ART.

**Results:**

The combined clinicopathological and radiomics model exhibited superior performance for the prediction of recurrence compared with the clinicopathological model in the training set (area under the curve [AUC] 0.78 vs. 0.67, P = 0.042), which was also validated in the test set (AUC 0.77 vs. 0.61, P = 0.192). In patients with a high probability of recurrence by the combined model, the 5-year progression-free survival was significantly improved with ART (92% vs. 57%, P = 0.024), and the median time to recurrence was longer (54 vs. 17 months after surgery).

**Conclusions:**

Radiomics significantly contributes added value in predicting recurrence when integrated with the clinicopathological features in patients with grade 2 meningiomas. Furthermore, the combined model can be applied to identify high-risk patients who require ART.

**Supplementary Information:**

The online version contains supplementary material available at 10.1186/s13014-022-02090-7.

## Introduction

Meningiomas are the most common primary intracranial neoplasms in adults, comprising 36.7% of all intracranial tumors [1]. Since the serial updates of the World Health Organization (WHO) grading classification, the proportion of grade 2 meningiomas has gradually increased up to 15–20% [2]. However, despite recent WHO grading scheme, there are limitations in predicting the prognosis of grade 2 meningiomas [3]. Grade 2 meningiomas are known to have an unpredictable heterogeneous disease course; even after gross total resection (GTR), recurrence can occur in a substantial number of patients [4, 5], whereas some patients experience a long indolent clinical course without adjuvant treatment [6].

Currently, adjuvant radiotherapy (ART) is recommended as a standard therapy for patients who undergo subtotal resection (STR) in grade 2 meningiomas [7] based on evidence indicating improvement in local control and survival rates with ART [8–10]. However, there is a lack of consensus on whether ART reduces the risk of tumor recurrence after GTR of grade 2 meningiomas [3]. Thus, it is crucial to establish a model for predicting a patient’s individual outcome and to identify high-risk patients who could benefit from ART even after GTR of grade 2 meningiomas. Patients who are at a low risk for recurrence can be spared from ART and its potential risks. Furthermore, in a set of patients who have undergone STR, patient subsets with a higher risk of recurrence may undergo more intensified radiotherapy to improve their respective outcomes.

Radiomics is an advanced technique that extracts high-dimensional quantitative imaging features, such as intensity distributions, spatial relationships, textural heterogeneity, and shape descriptors [11, 12]. Radiomics aims to discover meaningful “hidden” information within radiological images that is visually inaccessible. Previous studies have demonstrated the use of radiomics in differential diagnosis and grade prediction for meningiomas [13–15]. Some studies have also shown that radiomics can predict tumor recurrence in patients with meningiomas [16, 17]. We hypothesized that radiomics can enable risk stratification for tumor recurrence after surgery in patients with grade 2 meningiomas, which may guide patients toward ART.

Therefore, this study aimed to investigate whether radiomics features can be used to improve the prediction of tumor recurrence over clinicopathological features, and if radiomics can be used to identify high-risk patients who require ART in WHO grade 2 meningiomas.

## Methods

### Patient population

This study was approved by the institutional review board of the Yonsei University Health System (9-2021-0047). The institutional review board waived the requirement to obtain informed patient consent for this retrospective study. We retrospectively reviewed 199 patients with surgically confirmed WHO grade 2 meningiomas who underwent preoperative conventional magnetic resonance imaging (MRI) between February 2005 and November 2018. The exclusion criteria were as follows: (1) incomplete MRI sequences (n = 30), (2) suboptimal image quality (n = 6), (3) patients who received stereotactic radiotherapy (n = 5), and (4) lack of Ki-67 labeling index (n = 3). Finally, a total of 155 patients were enrolled (Additional file 1: Fig. S1). The study population was randomly divided into training and test sets with a ratio of 6:4 (n = 92 and n = 63, respectively).

Preoperative MRI was performed using a 3.0-T MRI scanner (Achieva, Philips Medical Systems, Amsterdam, Netherlands) with an eight-channel sensitivity-encoding head coil. Detailed parameters of the MRI sequences are provided in Additional file 1: Supplementary Material S1.

### Treatments

All patients underwent either GTR (n = 132) or STR (n = 23) surgery. The extent of resection was defined by comparing preoperative and postoperative MRI scans; GTR was defined by a lack of residual enhancing tumor in the image, and STR was defined by the patient having more than 50% of the tumor removed [18]. After surgical resection, a multidisciplinary team, consisting of neurosurgeons, radiation oncologists, neuropathologists, and neuroradiologists, decided whether to perform ART, which consisted of three-dimensional conformal radiotherapy and intensity-modulated radiotherapy. A total of 97 patients underwent ART; 80 patients received it after GTR (80/132, 60.6%), whereas 17 received it after STR (17/23, 73.9%). Among the patients who underwent ART, intensity-modulated radiotherapy was performed on 86 patients (88.7%, median 60.0 Gy), and three-dimensional conformal radiotherapy was performed on 11 patients (11.3%, median 59.4 Gy).

### Response assessment by a neuro-oncology meningioma working group

The evaluation of the tumor response and progression was determined according to the Response Assessment in Neuro-Oncology (RANO) criteria [19] by comparing serial MRIs of each patient. A radiation oncologist and neuroradiologist (with 9 and 8 years of experience, respectively) performed the evaluation and the results were achieved by consensus. A detailed description of the RANO criteria and the definitions of each assessment criteria are presented in the Additional file 1: Supplementary material S2 and Additional file 1: Table S1. The patients assessed as having a progressive disease in the follow-up periods were considered to have tumor recurrence. Patients assessed as having complete response, partial response, minor response, and stable disease were considered to be patients without tumor recurrence. The primary endpoint was tumor recurrence assessed by the RANO criteria during the follow-up period.

### Image preprocessing and radiomic feature extraction

Preprocessing of the T2 and T1C images was performed to standardize the data analysis across patients. Before analysis, the N4 bias correction algorithm was applied [20], and images were z-score normalized. Images were processed using an open-source software package (3D Slicer, version 4.11.0; available at: http://slicer.org/). T1C images were coregistered to T2 images via affine transformation with normalized mutual information as a cost function [21, 22]. The regions of interest were drawn on every tumor section on the T1C images using threshold-based and edge-based algorithms. Gross cystic, hemorrhagic, or necrotic areas of the tumors were included in the regions of interest (ROIs). The segmentations were transferred to the T2 images. A neuroradiologist (8 years of experience) performed segmentation which was confirmed by a second neuroradiologist (11 years of experience). Both neuroradiologists were blind to the corresponding clinical information.

**Fig. 1:**
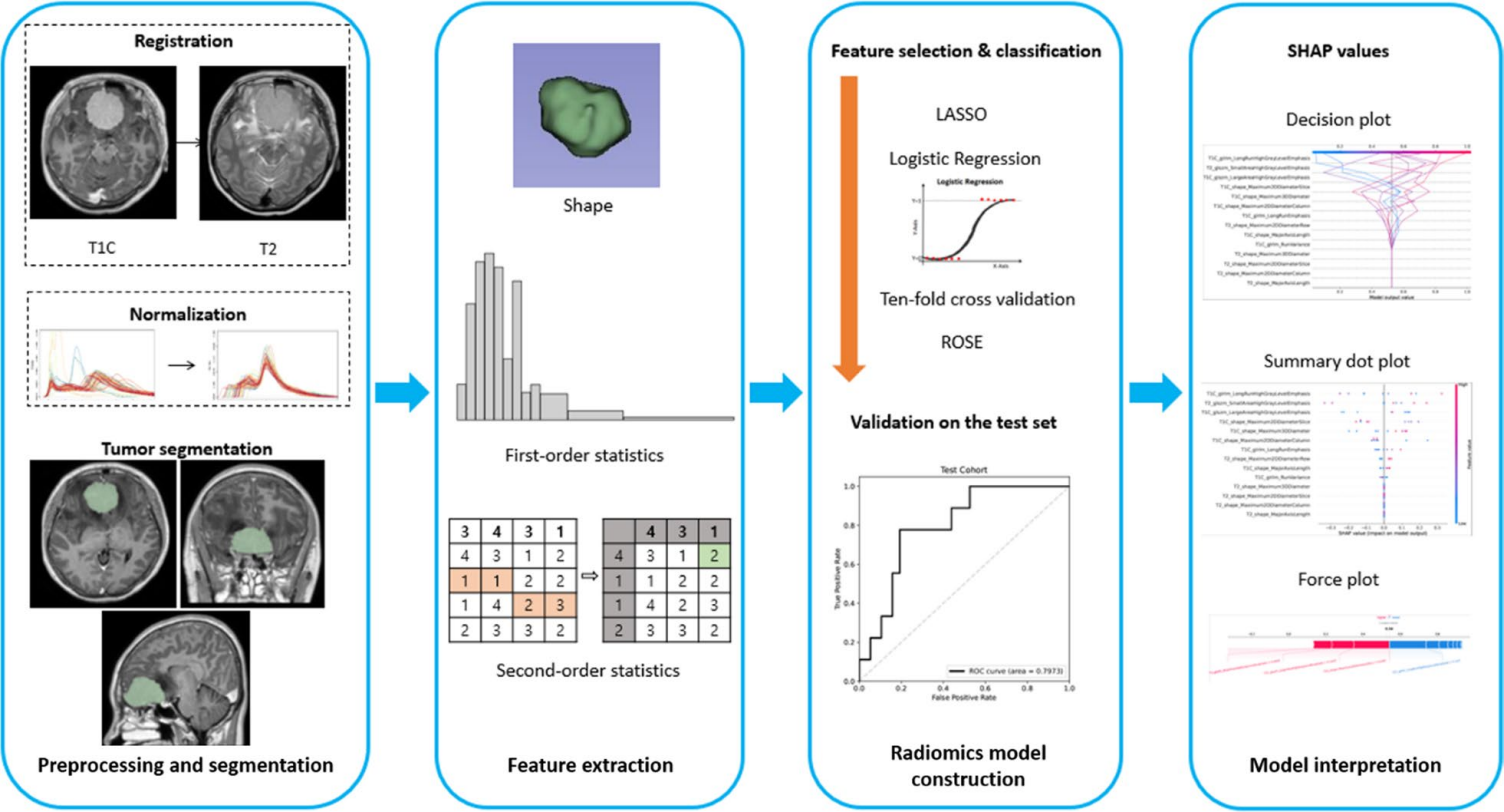
Workflow of image preprocessing, radiomics feature extraction, and machine learning.

Discretization using a fixed bin number of 32 was applied to extract the radiomic features [23] from the ROIs using an open-source Python-based module (PyRadiomics, version 2.0) [24], which adhered to the Image Biomarker Standardization Initiative [25]. Fourteen shape features, 18 first-order features, and 75 s-order features (24 Gy-level co-occurrence matrices, 16 Gy-level run length matrices, 16 Gy-level size zone matrices, 14 Gy-level dependence matrices, and 5 neighborhood gray-tone difference matrices) were extracted from the ROIs in the T1C and T2 images (Additional file 1: Table S2), comprising a total of 200 radiomic features. A schematic of the data processing is shown in Fig. 1.

### Model construction and comparison of diagnostic performance

The number of radiomic features was larger than the number of cases; therefore, the least absolute shrinkage and selection operator (LASSO) was applied to select the significant features, which optimized the feature space by removing both irrelevant and redundant features [18]. The base radiomics classifiers were constructed using logistic regression with tenfold cross validation. In addition, each model was trained using random over-sampling examples (ROSE) to overcome any data imbalance, and hyperparameters were Bayesian optimized. To evaluate whether radiomics improves the prediction of models, two models were trained as follows: (1) a clinicopathological model trained on clinical features, including age, extent of resection, ART status, and Ki-67 index; and (2) a combined model trained on clinicopathological and radiomics features. For statistical analysis, the Ki-67 index was dichotomized based on a cutoff value of 5% (≤ 5% vs. > 5%). Models were developed from the training set (n = 92) and validated on the test set (n = 63). The area under the receiver operating characteristic curve (AUC), accuracy, sensitivity, and specificity were obtained. The feature selection and machine learning process were performed using Python 3 (Python Software Foundation, Wilmington, Delaware, USA) with the Scikit-Learn library module (version 0.21.2). The performances of the models were compared based on the AUC using DeLong’s method [26].

### Model interpretability with SHapley Additive exPlanations

**Fig. 2:**
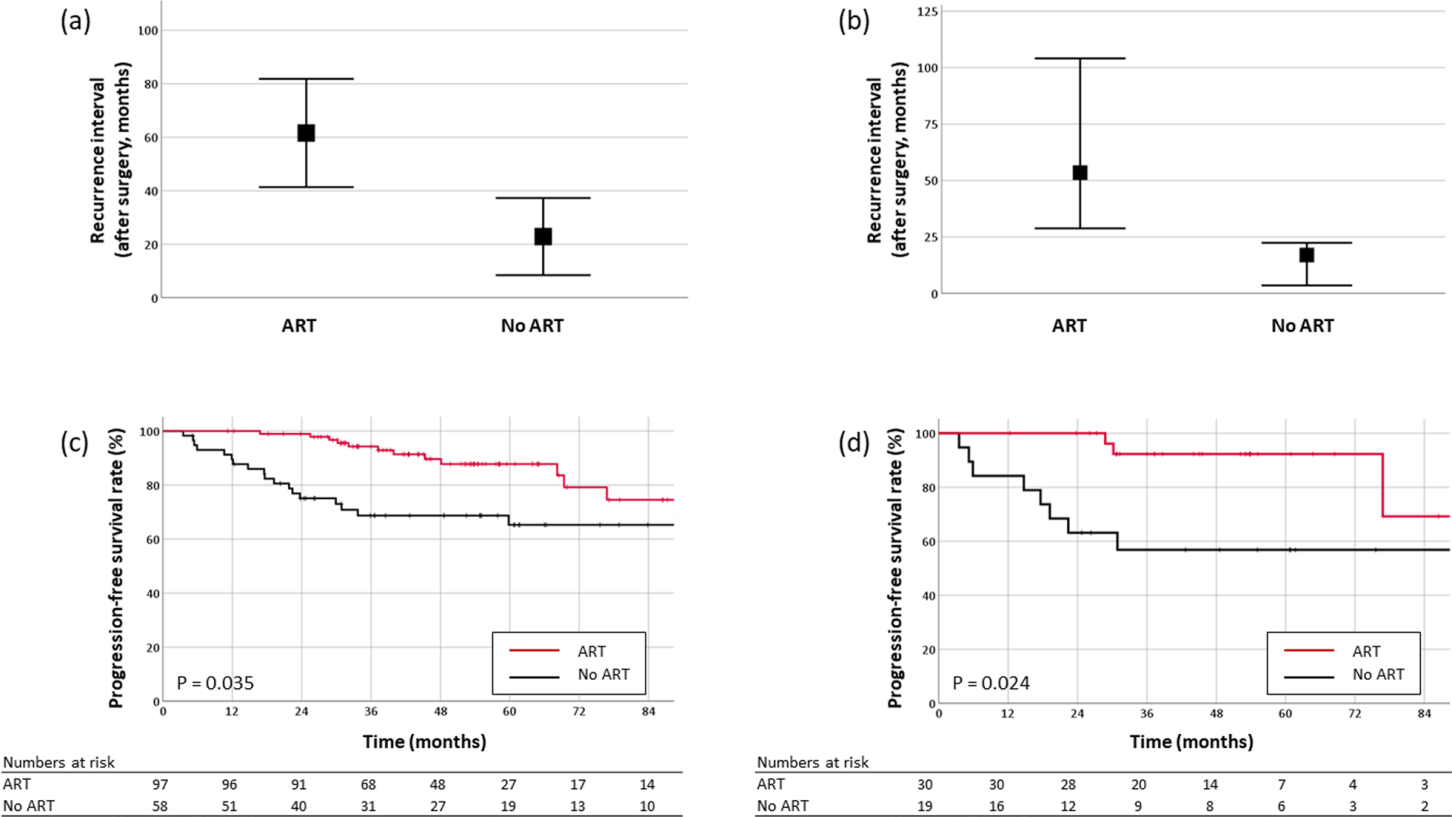
Comparison of median recurrence interval between patients with and without ART **a** in the entire cohort and **b** in high-risk patients of the test set (probability > 0.3, according to the combined clinicopathological and radiomics model). Kaplan–Meier curves of PFS **c** comparing patients with and without ART in the entire cohort and **d** comparing patient subgroups according to the utility of ART in high-risk patients. ART = adjuvant radiotherapy; PFS = progression-free survival. Data are presented as the median with a 95% confidence interval for each group

To interpret and analyze the radiomic features of the radiomics model, SHapley Additive exPlanations (SHAP), which is a game theoretic approach to explain the output of a tree-based machine learning model (Additional file 1: Supplementary Material S3), was applied [27]. SHAP measures the contribution of each feature of a model against the increase or decrease of the probability of a single output (i.e., the probability for tumor recurrence in our study) (Fig. 2).

### Analysis of survival outcomes and stratification for candidates for ART

After constructing the best combined model for predicting tumor recurrence, the probability value of each patient in the test set was analyzed to stratify candidates for ART. Youden’s index was used for the optimal cut-off selection threshold, and a cut-off probability value of 0.3 was obtained in the training set. The identical threshold value was applied to the test set, and patients were divided into low- and high-risk groups. We retrospectively analyzed whether patients in low- and high-risk groups benefited from ART by comparing the progression-free survival (PFS). PFS was defined as the time from initial surgery to tumor recurrence, death, or the last follow-up. Kaplan–Meier curves were generated and a log-rank test was performed to test the difference of PFS between patients who did and did not receive ART for each risk subgroup.

### Statistical analysis

The Student’s t-test, Mann–Whitney U-test, and Chi-square test were performed to compare patient characteristics between the responders and nonresponders from training and test sets. The Kaplan–Meier method was used to estimate survival rates, and the log-rank test was performed to compare survival between the two groups. A P-value < 0.05 was considered statistically significant. All statistical analyses were performed using statistical software R (version 4.0.1; R Foundation for Statistical Computing, Vienna, Austria) and SPSS (version 25.0; SPSS Inc., Chicago, IL).

## Results

### Patient characteristics

Among 155 patients (mean age 56.9 ± 14.3, 102 females and 53 males), 148 had atypical meningiomas, 6 had choroid meningioma, and 1 had clear cell meningioma. In the training set, GTR was more frequently performed in patients without tumor recurrence compared to those with tumor recurrence (87.5% vs. 58.3%, P = 0.011). No other clinicopathological features were different between patients with and without tumor recurrence. In the test set, GTR was more frequently performed in patients without tumor recurrence compared to those with tumor recurrence (90.7% vs. 66.7%, P = 0.045). Patients with tumor recurrence were significantly older (P = 0.020), showed a male predominance (P = 0.031), and had a higher Ki-67 labeling index (≥ 5%) (P = 0.018). ART was performed in 56 (60.9%) and 41 patients (65.1%) in the training and test sets, respectively. There were no significant differences between the training and test sets with respect to the clinical and pathologic variables. The clinicopathological characteristics of the training and test sets are summarized in Table 1.

**Table 1:** Patient characteristics in the training and test sets

Clinical variables	Training set (n = 92)		P-value^a^	Test set (n = 63)		P-value^a^	P-value^b^
	Without tumor recurrence	With tumor recurrence		Without tumor recurrence	With tumor recurrence		
	(n = 80)	(n = 12)		(n = 54)	(n = 9)		
Age (years)	57.3 ± 14.6	54.4 ± 13.6	0.530	55.7 ± 14.1	67.7 ± 13.2	0.020	0.838
Female ratio	52 (65.0%)	9 (75.0%)	0.494	38 (70.4%)	3 (33.3%)	0.031	0.875
Extent of resection			0.011			0.045	0.535
GTR	70 (87.5%)	7 (58.3%)		49 (90.7%)	6 (66.7%)		
STR	10 (12.5%)	5 (41.7%)		5 (9.3%)	3 (33.3%)		
Ki-67 labeling index	6.03 ± 4.81	6.96 ± 2.73	0.515	5.9 ± 4.3	8.2 ± 3.1	0.131	0.874
< 5%	32 (40.0%)	3 (25.0%)	0.076	29 (53.7%)	1 (11.1%)	0.018	
≥ 5%	38 (47.5%)	9 (75.0%)		25 (46.3%)	8 (88.9%)		
ART			0.659			0.517	0.595
Performed	48 (60.0%)	8 (66.7%)		36 (66.7%)	5 (55.6%)		
Not performed	32 (40.0%)	4 (33.3%)		18 (33.3%)	4 (44.4%)		
ART modality^c^			0.851			0.061	0.813
3D-CRT	5 (10.4%)	2 (25.0%)		2 (5.6%)	2 (40.0%)		
IMRT	43 (89.6%)	6 (75.0%)		34 (94.4%)	3 (60.0%)		
ART dose (Gy)^c^	57.3 ± 4.0	57.2 ± 6.7	0.956	58.3 ± 3.9	58.7 ± 2.6	0.853	0.204

Data are expressed as the mean with standard deviation in parentheses, median with interquartile range in parentheses, or number with percentage in parentheses

^a^Calculated from Student’s-t test for continuous variables and Chi-square test for categorical variables to compare the patient characteristics between the responder and non-responders from each training and test set

^b^Calculated from Student’s-t test for continuous variables and Chi-square test for categorical variables for the comparison of training and test sets

^c^Data obtained from patients who underwent adjuvant radiotherapy following surgery

GTR: gross total resection; STR: subtotal resection; ART: adjuvant radiotherapy; RT: radiotherapy; 3D-CRT: three-dimensional conformal radiotherapy; IMRT: intensity modulated radiotherapy

### Treatment outcomes and prognostic factors

With a median follow-up of 63.8 months (range, 6.6–190.7 months), there were 21 patients with tumor recurrence (12 [13.0%] in the training set, and 9 [14.3%] in the test set). Fifteen events occurred within 24 months (7 in the training set, and 8 in the test set, P = 0.292). The 5-year PFS rate was 79.1% for the entire cohort, and 79.9% and 78.9% for the training and test set, respectively (P = 0.529).

In the training set, tumor recurrences occurred more frequently in patients who underwent STR than in those who underwent GTR (33.3% vs. 9.1%, P = 0.011). The recurrence rate was higher in patients with a Ki-67 index value of 5% or more, although it was not statistically significant (19.1% vs. 6.7%, P = 0.076). In the test set, the recurrence rate was higher in patients who underwent STR (37.5% vs. 10.9%, P = 0.045) and in patients with Ki-67 ≥ 5% (24.2% vs. 3.3%, P = 0.018).

### Radiomics model construction of recurrences and comparison of diagnostic performance

In the clinicopathological model, the AUC, accuracy, sensitivity, and specificity were 0.67 (95% confidence interval [CI] 0.60–0.74), 66.9%, 48.2%, and 85.3% in the training set, respectively, and 0.61 (95% CI 0.44–0.78), 81.3%, 33.3%, and 89.1%, respectively, in the test set.

In the combined clinicopathological and radiomics model, a total of seven features were selected: two clinical features (extent of resection [GTR or STR] and Ki-67 index [≥ 5% or < 5%]) and five radiomic features, which were all first-order features from T1C (10^th^ percentile, 90^th^ percentile, entropy, mean absolute deviation, and minimum) (Additional file 1: Table S3). The AUC, accuracy, sensitivity, and specificity were 0.78 (95% CI 0.70–0.85), 75.0%, 76.8%, and 73.1% in the training set, and 0.77 (95% CI 0.60–0.94), 70.3%, 66.7%, and 70.9%, respectively, in the test set.

**Fig. 3:**
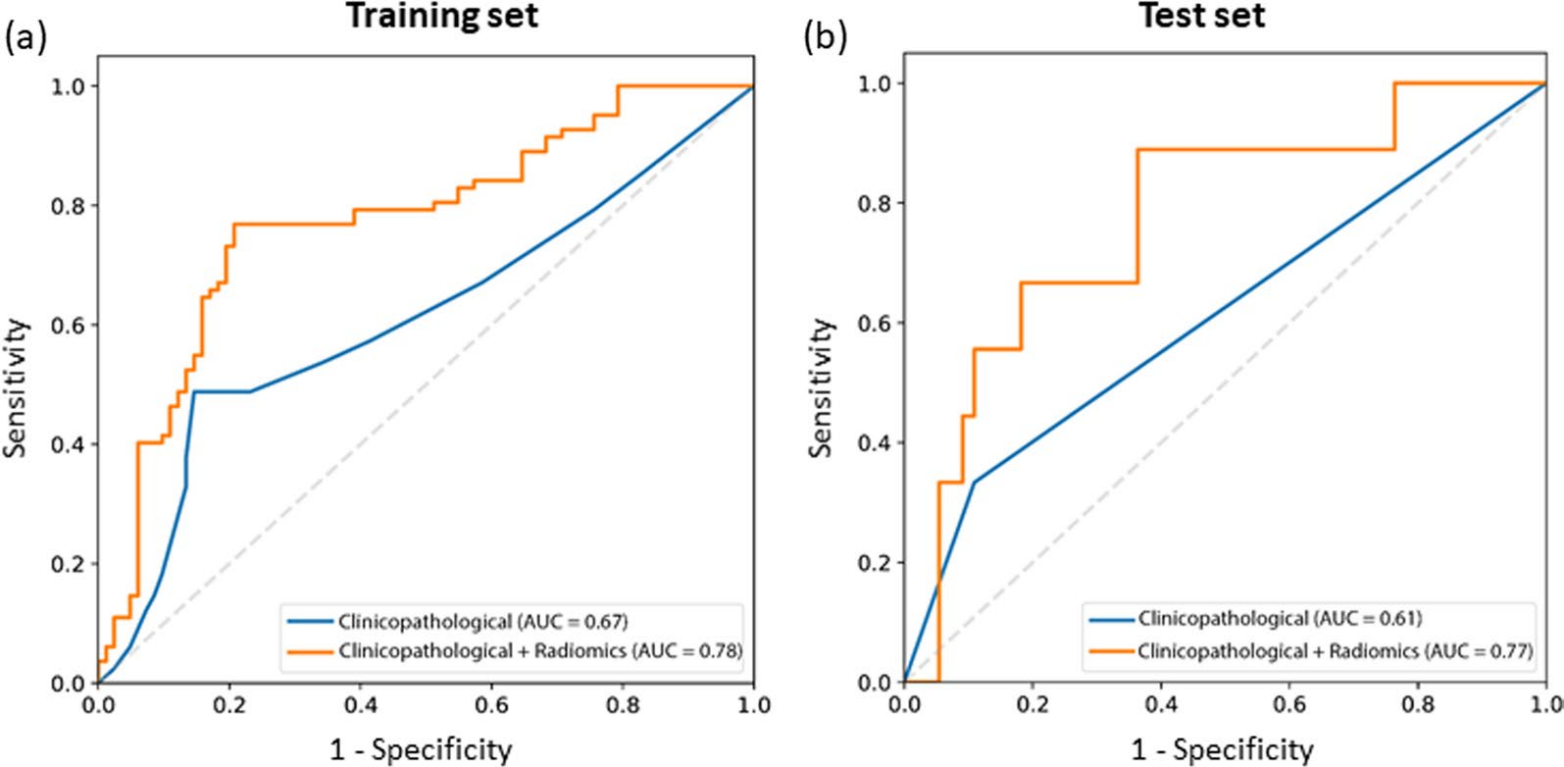
Receiver operating characteristic curves of the radiomics model in the **a** training and **b** test sets

In the training set, the combined model achieved superior performance compared with the clinicopathological model (AUC: 0.78 vs. 0.67, P = 0.042) (Fig. 3, Table 2). In the test set, the combined model trended toward better performance in the test set than in the clinicopathological model (AUC: 0.77 vs. 0.61, P = 0.192) without statistical significance. The diagnostic performances of the two models in the training and test set are provided in Table 2.

**Table 2:** Performances of machine learning models for prediction of tumor recurrence in the training and test set

Models	Training set					Test set				
	AUC (95% CI)	Accuracy (%)	Sensitivity (%)	Specificity (%)	P value	AUC (95% CI)	Accuracy (%)	Sensitivity (%)	Specificity (%)	P value
Clinicopathological model	0.67 (0.59–0.74)	66.9	48.2	85.3	Reference	0.61 (0.44–0.78)	81.3	33.3	89.1	Reference
Clinicopathological + radiomics model	0.78 (0.70–0.85)	75.0	76.8	31.0	0.042	0.77 (0.60–0.94)	70.3	66.7	70.9	0.192

AUC: area under the curve; CI: confidence interval; NRI: net reclassification index

### Model interpretability with SHAP

**Fig. 4:**
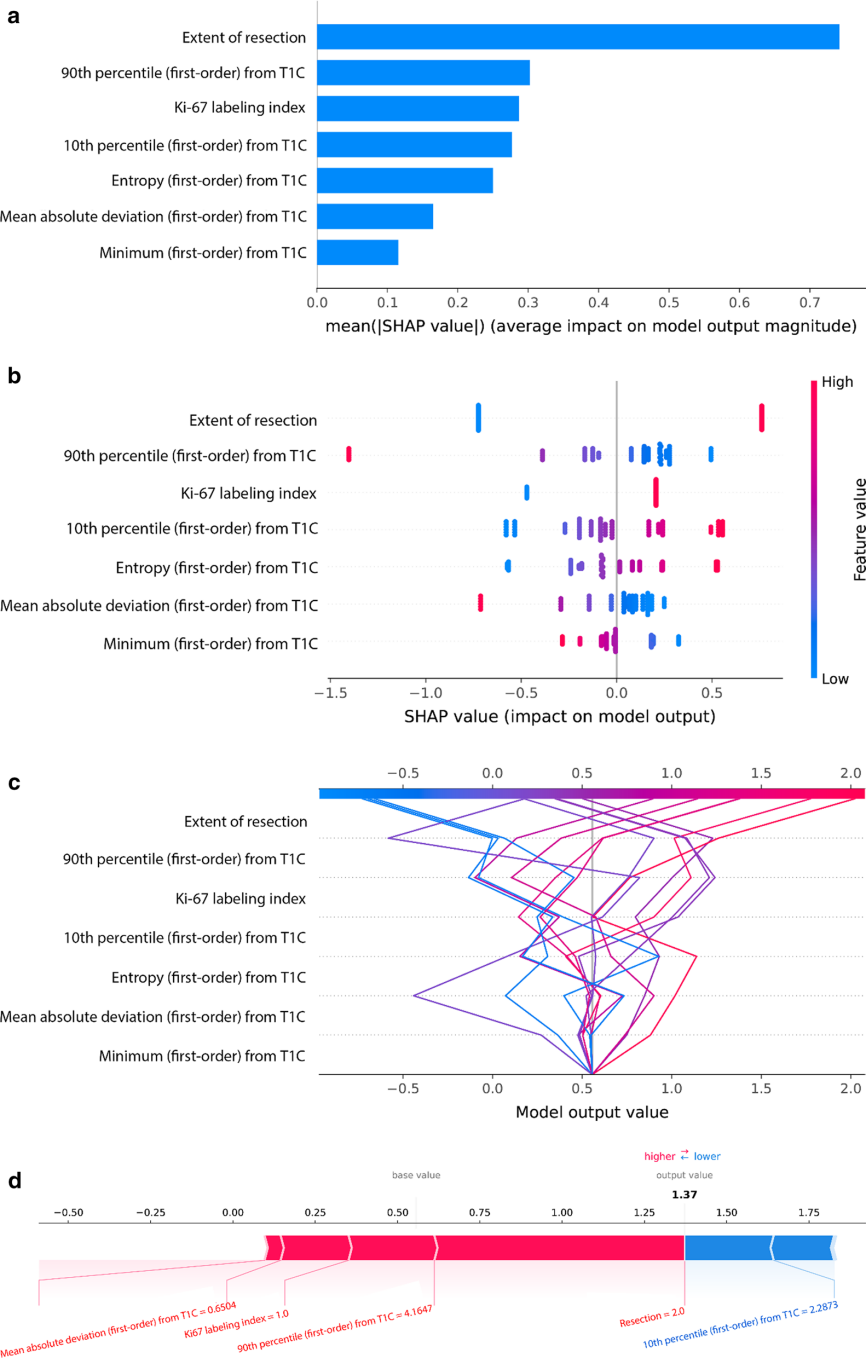
Model interpretability of a combined clinicopathological and radiomics model for the prediction of tumor recurrence with SHAP in the training set. **a** Variance importance plot that lists the most significant variables in descending order. **b** Summary plot of feature impact on the decision of the model and interaction between the features in the model. A positive SHAP value indicates an increase in the probability of tumor recurrence. **c** Decision plot showing how the model predicts tumor recurrence. Starting at the bottom of the plot, the prediction line shows how the SHAP values accumulate from the base value to arrive at the model’s final score at the top of the plot and how each feature contributes to the overall prediction of tumor recurrence. **d** Force plot of a representative case of a patient with tumor recurrence. Red arrows represent feature effects that drive the prediction value higher, and blue arrows are those effects that drive the prediction value lower. Each arrow’s size represents the magnitude of the corresponding feature’s effect. Note that the extent of resection, 90th percentile from T1C, and Ki-67 labeling index largely push the model prediction score higher than the base value

The SHAP values for each selected feature in the combined clinicopathological and radiomics model were calculated. The variance importance plot, summary plot, decision plot, and force plot of the test set are shown in Fig. 4. For each prediction, a positive SHAP value indicates an increase in the risk of tumor recurrence while a negative SHAP value indicates reduced risk. As observed in the plots, the extent of resection, 90th percentile from T1C, and Ki-67 index are the three most important risk factors.

### Selection of candidates for ART using the developed combined model

In the test set, there were 49 patients in the high-risk group and 14 patients in the low-risk group according to the combined clinicopathological and radiomics model. The characteristics of patients in the high- and low-risk groups are summarized in Additional file 1: Table S4. Among all patients, those who received ART exhibited a significantly longer PFS (5-year PFS, 87.8% vs. 65.4%, P = 0.035) and delayed recurrence (median time to recurrence 45.3 vs. 18.4 months) (Fig. 2a, c). In the high-risk group, the PFS was significantly longer in patients who received ART (5-year PFS, 92.3% vs. 56.8%, P = 0.024) (Fig. 2b), with significantly delayed recurrence (median 53.5 vs. 17.0 months). In the low-risk group, there was no significant difference in the PFS (P = 0.264) or recurrence interval with respect to ART. Only one patient experienced tumor recurrence (39.9 months after surgery).

When the cohort of patients was divided into four groups according to ART status and recurrence probability, the PFS was observed to significantly improve after ART in patients with a high recurrence probability (median 8.7 year, 5-year 92.3%) (Fig. 2d). The significant improvement in PFS was observed regardless of the extent of resection (GTR: 5-year 91.3% vs. 63.5%, P = 0.054; STR: 5-year 100.0% vs. 0.0%, P = 0.012).

## Discussion

In this study, radiomic features derived from preoperative MRI were applied to predict tumor recurrence in grade 2 meningiomas. The model incorporating radiomic features with clinicopathologic features exhibited significantly a higher prediction accuracy for tumor recurrence. Several risk factors for tumor recurrence were identified using SHAP, and the contribution of each feature to the probability of tumor recurrence was determined. Further, radiomics enabled a subset of patients at a high risk for tumor recurrence to be identified, and we observed that the high-risk patients clearly benefitted from ART. Therefore, radiomics can serve as a potential imaging biomarker, as well as a useful tool for selecting adequate candidates for ART in patients with meningiomas.

Although the majority of meningiomas are benign and slow growing, WHO grade 2 meningiomas are considered to have a high recurrence rate of 55% and low survival rates [28–30]. ART after the STR of grade 2 meningiomas is widely practiced; however, the impact of ART on grade 2 meningiomas after a GTR remains contentious. Several researchers demonstrated improved local control and/or survival [8–10, 30–33] with ART after GTR, but a subset exhibited contrary results after ART [9, 34–36]. According to two multi-institutional phase II studies (NRG Oncology RTOG 0539 [32] and EORTC 22,042–26,042 [37]), which enrolled grade 2 meningioma patients who underwent GTR and ART, there is a potential survival benefit with a 3-year PFS of 89%–94%. However, those studies did not directly compare ART with upfront observation, and risk factors other than surgical resection were not addressed. Therefore, a uniform treatment paradigm for resected grade 2 meningiomas has yet to be established.

Because there is a lack of standardized treatment for resected grade 2 meningiomas, performing noninvasive risk stratification prior to adjuvant therapies is highly desirable. Even after surgical resection, patients at high-risk for tumor recurrence can be considered for intensified adjuvant therapies, and patients at low-risk for tumor recurrence can be spared from experiencing the possible neurotoxicities of ART [38]. Several previous studies investigated clinical prognostic factors for grade 2 meningiomas, which included age, sex, extent of resection, tumor invasiveness (i.e., brain or bone invasion), and higher MIB-1 labeling index [39, 40]. Similar to our study, the clinical significance of the Ki-67 labeling index on local control and survival in high-grade meningiomas has been widely reported. A higher Ki-67 labeling index seems to be related to invasiveness, which is closely correlated with an incomplete resection rate. Although there is some discrepancy in the exact cutoff point, several studies have suggested the need for ART in patients with a higher Ki-67 [41, 42]. Pretreatment tumor size or volume was also reported as a significant predictor of prognosis [35, 43–45]. However, in our study that analyzed various radiomic features including tumor size, it was not a significant prognostic factor compared to other features.

Several studies attempted to identify the prognostic value of radiomics in patients with meningiomas [16, 17, 46]. However, these previous studies combined grade 1–3 meningiomas and do not provide specific information on the utility of the radiomics model for grade 2 meningiomas. Moreover, neither the combined clinicopathological model nor radiomics model showed any improved performance in predicting prognosis compared with the clinicopathological model alone, limiting the real-world application of the radiomics model [17, 46]. Decision-making for the treatment of grade 2 meningioma patients depends on multilevel prognostic information and clinicopathological information, such as the extent of the tumor resection or Ki-67 index; therefore, we focused on the role of radiomics in predicting survival given the multilevel prognostic information. Our results show that radiomics significantly increases the model performance when added to a clinicopathological model, thus promoting the integration of radiomics in clinical practice.

Radiomics models have limited explainability because they rely on complex machine learning algorithms, resulting in low clinical utility [47]. SHAP can uncover complex underlying patterns [27, 48] and has recently been utilized to interpret radiomics models [49, 50]. This analysis identified two clinical features—the extent of resection and the Ki-67 labeling index—and five radiomic first-order features from T1C that contributed the most towards the prediction of tumor recurrence. The extent of resection and the Ki-67 index are well-known prognostic factors for tumor recurrence [35, 51], which was also verified by our study. The 90^th^ and 10^th^ percentile values from T1C, which are the two most important radiomic features, denote the intensity of contrast enhancement. Heterogeneous contrast enhancement or the presence of contrast enhancement of meningiomas [17] has shown significant association with high-grade meningiomas or tumor invasiveness [52, 53]; therefore, the meningioma grade may be indicated by the percentile-related first-order features, which reflect the distribution of the contrast-enhancement degree.

Our study has several limitations. First, our study was based on a single-center, retrospectively collected dataset. Further studies with a larger number of patients and external validations are required. Second, the histopathologic risk factors, such as the MIB-1 labeling index or mitoses index, were not evaluated in our study because relevant information for the majority of patients were unavailable during the long study-inclusion period. To prove the predictive values of radiomics, it is highly desirable to incorporate detailed histopathologic features into the models in future studies. Third, the probability scores derived from the developed model for tumor recurrence were only calculated in the test set, not in the training set, because the training set was oversampled during the development of the model. Therefore, the benefit of ART was only evaluated in the test set, which inevitably decreased the sample size. However, based on our study results, we at least found a benefit of ART in a subgroup of the test set, which was identified through the combined model. Future studies with larger sample sizes are required to identify the patients with grade 2 meningiomas who might benefit from ART.

Our study is the first to show the possibility of radiomics playing an important role in selecting candidate patients with meningiomas for ART. Over a similar follow-up period (median 54 months in ART group vs. 61 months in non-ART group, P = 0.584), we observed that ART prolongs PFS and significantly delays recurrence in high-risk patients. Our combined predictive model could be applied effectively and conveniently in both GTR and STR patients.

## Conclusions

Multiparametric MRI radiomics has an added prognostic value for the prediction of tumor recurrence when integrated with clinicopathologic profiles in patients with grade 2 meningiomas. With radiomics, we could identify a subset of patients at a high risk for tumor recurrence who might benefit from intensified treatment. Therefore, radiomics may be used as a potential imaging biomarker in patients with grade 2 meningiomas.

## Supplementary Information

**Additional file 1.** Supplementary figures and tables.

## Data Availability

Research data are stored in an institutional repository and will be shared upon request to the corresponding author.

## Abbreviations

ART: Adjuvant radiotherapy
GTR: Gross total resection
LASSO: Least absolute shrinkage and selection operator
PFS: Progression-free survival
RANO: Response assessment in neuro-oncology
ROI: Region of interest
ROSE: Random over-sampling examples
SHAP: Shapley Additive Explanations
STR: Subtotal resection
T1C: Post-contrast T1-weighted image
T2: T2-weighted image
WHO: World Health Organization
