# Supplementary material for: An interpretable radiomics model to select patients for radiotherapy after surgery for WHO grade 2 meningiomas
Source: Radiat Oncol. 2022 Aug 22;17:147. doi: 10.1186/s13014-022-02090-7 (PMC9396861; doi:10.1186/s13014-022-02090-7)
Supplement: Supplementary file 1 — Additional file 1. Supplementary figures and tables. [file 13014_2022_2090_MOESM1_ESM.docx]

**Supplementary Data**

**Supplementary Figure 1. Flow chart of patient enrollment**

**
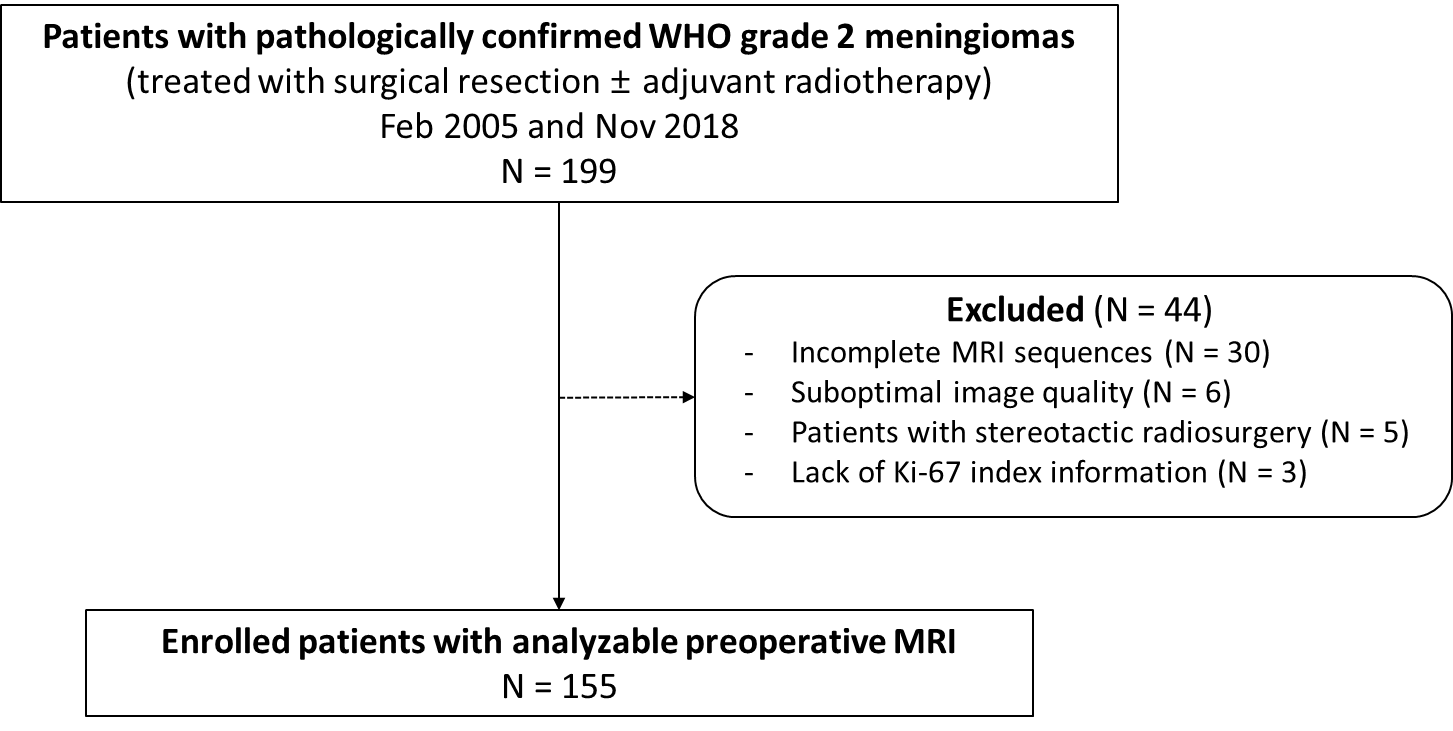
**

**Supplementary Material S1. MRI Protocol**

A preoperative MRI was performed using a 3.0-T MRI scanner (Achieva, Philips Medical Systems, Amsterdam, Netherlands) with an eight-channel sensitivity-encoding head coil. The preoperative MRI protocol included T1-weighted (TR/TE 2000/10 ms; field of view, 230 mm; section thickness, 5 mm; matrix, 320 × 198); and T2-weighed (T2) images (TR/TE 3000-9000/80-120 ms; field of view, 230 mm; section thickness, 3–5 mm; matrix, 320 × 198). Postcontrast T1-weighted (T1C) (TR/TE, 2000/10 ms; field of view, 250 mm; section thickness, 1–2 mm; and matrix, 256 × 256) were acquired after the administration of 0.1 mL/kg of gadolinium-based contrast material (Gadovist; Bayer).

**Supplementary Material S2. RANO criteria for meningioma assessment**

The evaluation of the tumor response and progression was determined according to newly proposed RANO criteria [1] by comparing serial MRIs of each patient. The evaluation of the tumor size was based on the product of the maximal cross-sectional enhancing diameters. A measurable disease was defined as bidimensional contrast enhancing lesions with clearly defined margins in an MRI. Additionally, it has two perpendicular diameters of at least 10 mm, and it is visible on two or more axial slices that have a preferable interslice thickness of 5 mm at most, with an interslice gap no larger than 1 mm. A nonmeasurable disease was defined as lesions with maximal perpendicular diameter measures that are less than 10 mm, those that are measurable in one dimension only, or masses with ill-defined margins. The radiographic response was determined in comparison with the tumor measurement obtained at the pretreatment baseline to determine the response, and the smallest tumor measurement at either the pretreatment baseline or after the initiation of therapy was used to determine the progression.

All baseline evaluations were performed not more than four weeks before the beginning of treatment. A sum of the products of the maximal perpendicular diameters for all target lesions, which should be measurable, was calculated and reported as the baseline area. In cases of multiple lesions, at least two, and up to five, lesions were selected as target lesions. All other lesions were identified as nontarget lesions. The following table (Supplementary Table 1) lists the criteria for radiographic changes after therapy.

**Supplementary Material S3. Model interpretability with SHapley Additive exPlanations (SHAP)**

SHAP, originating from game theory, assesses the contribution of each variable of the model to its output [2, 3]. The output of each possible combination of other variables is collected. SHAP analysis enables the quantification of continuous and categorical variables in the texture features only and the combined models. Features listed higher on the left vertical axis indicate a stronger influence on the overall model outcome. Feature values are color coded: red data points indicate higher values, and blue data points indicate lower values  [4]. In addition, this allows the quantification of the impact of each variable on the prediction, not only on a global level (on the overall population) but also locally (on a subset or one patient)  [5]. Thus, Shapley values for each variable are additive, which makes the contribution of each variable convertible to a share of the output classification probability. This provides an intuitive visualization for clinicians using this model.

**Supplementary Table 1. Summary of the proposed RANO response criteria for meningiomas [1]**

| Criteria | CR | PR | MR | SD | PD |
| --- | --- | --- | --- | --- | --- |
| Target lesions | None | ≥50% decrease in area relative to  baseline | ≥25% and <50% decrease  in area relative  to baseline | <25% decrease relative  to baseline but <25%  increase in area relative  to nadir | ≥25% increase in area  relative to nadir* |
| Nontarget lesions | None | Stable or improved | Stable or improved | Stable or improved | Unequivocal PD* |
| New lesions | None | None | None | None | Present* |
| Corticosteroids | None | Stable or decreased | Stable or decreased | Stable or decreased | Not applicable |
| Clinical status | Stable or improved | Stable or improved | Stable or improved | Stable or improved | Worse* |
| Requirement for response | All | All | All | All | Any |

*Progression occurs when this criterion is met.

CR = Complete response; PR = Partial response; MR = Minor response; SD = Stable disease; PD = Progressive disease

Specific considerations were made for patients undergoing adjuvant radiotherapy  [1]. Radiotherapy may cause local inflammation; therefore, it may lead to the enlargement of target and nontarget lesions, which may not necessarily indicate tumor progression. In equivocal cases for which the distinction of treatment-related change and progression was not possible, the reviewers referred to the serial follow-up MRIs and concluded the final assessment. A consensus was reached between the two reviewers in cases of disagreement. In addition, unequivocal clinical deterioration was also regarded as a progressive disease.

**Supplementary Table 2. List of extracted radiomics features.**

| Feature category | Feature list |
| --- | --- |
| Shape (n = 14) | Volume, surface area, surface-area-to-volume ratio, sphericity, maximum 3D diameter, maximum 2D diameter (column), maximum 2D diameter (row), maximum 2D diameter (slice), major axis, minor axis, least axis, elongation, flatness, mesh volume |
| First order statistics (n = 18) | Energy, total energy, entropy, minimum, 10^th^ percentile, 90^th^ percentile, maximum, mean, median, interquartile range, range, mean absolute deviation, robust mean absolute deviation, root mean squared, skewness, kurtosis, variance, uniformity |
| GLCM (n = 24) | Autocorrelation, cluster prominence, cluster shade, cluster tendency, contrast, correlation, difference average, difference entropy, difference variance, inverse difference, inverse difference moment, inverse difference moment normalized, inverse difference normalized, informal measure of correlation 1, informal measure of correlation 2, inverse variance, joint average, joint energy, joint entropy, maximal correlation coefficient, maximum probability, sum average, sum entropy, sum of squares |
| GLRLM (n = 16) | Short-run emphasis, long-run emphasis, gray level nonuniformity, gray level nonuniformity normalized, run-length nonuniformity, run-length nonuniformity normalized, run percentage, gray level variance, run variance, run entropy, low gray level run emphasis, high gray level run emphasis, short-run low gray level emphasis, short-run high gray level emphasis, long-run low gray level emphasis, long-run high-gray level emphasis |
| GLSZM (n = 16) | Small area emphasis, large area emphasis, gray level non-uniformity, gray level non-uniformity normalized, size-zone non-uniformity, size-zone non-uniformity normalized, zone percentage, gray level variance, zone variance, zone entropy, low gray level zone emphasis, high gray level zone emphasis, small area low gray level emphasis, small area high gray level emphasis, large area low gray level emphasis, large area high gray level emphasis |
| GLDM (n = 14) | Small dependence emphasis, large dependence emphasis, gray level non-uniformity, dependence non-uniformity, dependence non-uniformity normalized, gray level 0076ariance, dependence variance, dependence entropy, low gray level emphasis, high gray level emphasis, small dependence low gray level emphasis, small dependence high gray level emphasis, large dependence low gray level emphasis, large dependence high gray level emphasis, |
| NTGDM (n = 5) | Coarseness, complexity, strength, contrast, busyness |

GLCM = gray level co-occurrence matrix, GLDM = gray level dependence matrix, GLRLM = gray level run-length matrix, GLSZM = gray level size zone matrix, NTGDM = neighboring gray tone difference matrix

**Supplementary Table 3. Seven selected features that were significantly associated with tumor recurrence in the combined clinicopathological and radiomics model**

|  | **Selected features** |
| --- | --- |
| Clinicopathological features (2) | Extent of resection  Ki-67 labeling index (≥5% vs. <5%) |
| Radiomic features (5) | 10^th^ percentile (first-order) from T1C  90^th^ percentile (first-order) from T1C  Entropy (first-order) from T1C  Mean absolute deviation (first-order) from T1C  Minimum (first-order) from T1C |

T1C = Post-contrast T1-weighted image

**Supplementary Table 4. Characteristics of the patients with a high recurrence probability and patients with a low recurrence probability**

|  |  |  | High risk patients  (n = 49) |  | Low risk patients  (n = 14) |  |  |  |
| --- | --- | --- | --- | --- | --- | --- | --- | --- |
|  |  |  | No. (%) |  | No. (%) |  | P value |  |
|  | Age (years) |  | 59.2±14.9 |  | 51.2±11.7 |  | 0.070 |  |
|  | Sex |  |  |  |  |  | 0.572 |  |
|  | Male |  | 18 (36.7%) |  | 4 (28.6%) |  |  |  |
|  | Female |  | 31 (63.3%) |  | 10 (71.4%) |  |  |  |
|  | Extent of resection |  |  |  |  |  | 0.479 |  |
|  | GTR |  | 42 (85.7%) |  | 13 (92.9%) |  |  |  |
|  | STR |  | 7 (14.3%) |  | 1 (7.1%) |  |  |  |
|  | Ki-67 labeling index |  |  |  |  |  | 0.009 |  |
|  | <5% |  | 19 (38.8%) |  | 11 (78.6%) |  |  |  |
|  | ≥5% |  | 30 (61.2%) |  | 3 (21.4%) |  |  |  |
|  | ART |  |  |  |  |  | 0.230 |  |
|  | Performed |  | 30 (61.2%) |  | 11 (78.6%) |  |  |  |
|  | Not |  | 19 (38.8%) |  | 3 (21.4%) |  |  |  |

GTR = gross total resection; STR = subtotal resection; ART = adjuvant radiotherapy

**References**

1. Huang RY, Bi WL, Weller M, Kaley T, Blakeley J, Dunn I, et al. Proposed response assessment and endpoints for meningioma clinical trials: report from the Response Assessment in Neuro-Oncology Working Group. Neuro Oncol. 2019;21(1):26-36.

2. Molnar C. Interpretable Machine Learning. Accessed on 17 June 2020. <https://christophm.github.io/interpretable-ml-book/shapley.html>.

3. Lundberg SM, Lee S-I. A unified approach to interpreting model predictions. In. Proceedings of the 31st international conference on neural information processing systems; 2017. p. 4768-77.

4. Awe AM, Vanden Heuvel MM, Yuan T, Rendell VR, Shen M, Kampani A, et al. Machine learning principles applied to CT radiomics to predict mucinous pancreatic cysts. Abdominal Radiology. 2021. <https://doi.org/10.1007/s00261-021-03289-0>.

5. Giraud P, Giraud P, Nicolas E, Boisselier P, Alfonsi M, Rives M, et al. Interpretable Machine Learning Model for Locoregional Relapse Prediction in Oropharyngeal Cancers. Cancers (Basel). 2020;13(1).
